# 3D ultrasound guided navigation system with hybrid image fusion

**DOI:** 10.1038/s41598-021-86848-1

**Published:** 2021-04-23

**Authors:** David Iommi, Alejandra Valladares, Michael Figl, Marko Grahovac, Gabor Fichtinger, Johann Hummel

**Affiliations:** 1 Center for Medical Physics and Biomedical Engineering, Medical University of Vienna, Spitalgasse 23, Vienna, 1090 Austria; 2 Division of Nuclear Medicine, Department of Biomedical Imaging and Image-Guided Therapy, Medical University of Vienna, Währinger Gürtel 18-20, Vienna, 1090 Austria; 3 Queen’s University, School of Computing, 25 Union St, 557 Goodwin Hall, Kingston, ON K7L 3N6 Canada

**Keywords:** Preclinical research, Scientific data

## Abstract

A prototype of a navigation system to fuse two image modalities is presented. The standard inter-modality registration is replaced with a tracker-based image registration of calibrated imaging devices. Intra-procedure transrectal US (TRUS) images were merged with pre-procedure magnetic resonance (MR) images for prostate biopsy. The registration between MR and TRUS images was performed by an additional abdominal 3D-US (ab-3D-US), which enables replacing the inter-modal MR/TRUS registration by an intra-modal ab-3D-US/3D-TRUS registration. Calibration procedures were carried out using an optical tracking system (OTS) for the pre-procedure image fusion of the ab-3D-US with the MR. Inter-modal ab-3D-US/MR image fusion was evaluated using a multi-cone phantom for the target registration error (TRE) and a prostate phantom for the Dice score and the Hausdorff distance of lesions . Finally, the pre-procedure ab- 3D-US was registered with the TRUS images and the errors for the transformation from the MR to the TRUS were determined. The TRE of the ab-3D-US/MR image registration was 1.81 mm. The Dice-score and the Hausdorff distance for ab-3D-US and MR were found to be 0.67 and 3.19 mm. The Dice score and the Hausdorff distance for TRUS and MR were 0.67 and 3.18 mm. The hybrid navigation system showed sufficient accuracy for fusion guided biopsy procedures with prostate phantoms. The system might provide intra-procedure fusion for most US-guided biopsy and ablation interventions.

## Introduction

Multi-modal image registration spatially aligns medical images from different modalities in the same image coordinate space. Image fusion can help achieve a more accurate diagnosis and treatment by integrating complementary information from multi-modal images. Image registration is useful in minimally invasive procedures such as image-guided surgery, image-guided biopsy, and radiotherapy planning where diagnostic information from pre-operative images (Positron-emission tomography (PET), Magnetic resonance (MR) or Computed tomography (CT)) integrates with intra-procedural imaging (e.g., ultrasound (US))^1^.

Multi-modality image registration is challenging, as it is difficult to determine a robust metric for clinical use^2^. Standard pairwise intensity-based image registration techniques optimize image similarity metrics; mutual information is the most used pixel-based metric for multi-modal image registration and utilizes statistical information from different modalities^3^. However, different image acquisition methods may cause a statistical correlation between image structures that do not correspond to the same anatomical structures, violating the hypotheses of intensity-based similarity techniques.

The idea behind this work was to develop a real-time navigation system that replaces the (commonly used) inter-modal registration by an intra-modal 3D-US/3D-US registration and the use of an optical tracking system (OTS) to allow image fusion between two arbitrary image modalities. This should avoid well known difficulties arising from inter-modal registration. Such challenges are described for a large variety of modalities. For example, in multi-modality image-guided prostate biopsies, the registration between MR and transrectal-US (TRUS) images is difficult because of the poor signal to noise ratio and lack of well-defined features in US images and the inhomogeneous imaging resolutions. Therefore, surface-based approaches are commonly used to perform the registration task, using image segmentation to address the differences between two modalities^4,5^. Hu et al.^6^ introduced a convolutional neural network (CNN) that infer voxel-level transformation from higher-level correspondence information from anatomical labels. During the training, the CNN estimates the displacement fields to align multiple labeled corresponding structures for individual image pairs. The network uses only unlabelled image pairs as input for inference. However, surface-based registration methods are primarily influenced by the information extracted from voxels proximal to the boundary of the organ. For this reason, they cannot guarantee adequate voxel-to-voxel correspondence of internal structures. Furthermore, CNNs based on the information contained in anatomical labels rely on the availability of labelled data. Deep learning based techniques aimed to learn the similarity metric of two different image modalities , such as MR-US, may struggle due to potentially poor initial alignment.

US imaging is used also for biopsies and ablations procedures for hepatic lesions. US imaging has worse contrast than MRI, but the contrast of CT in the prostate is poorer compared to US^7^. Some hepatic tumors cannot be seen on B-mode images due to their location, their small size, or their echogenicity. In these situations, US-CT/MR registration has been proven to improve the feasibility of percutaneous procedures^8,9^. Heinrich et al.^10^ proposed a local self-similarity based metric for inter-modal registration, which uses the similarity of small patches in one image modality to estimate a local representation of image structure. This metric may work to fuse CT with US but is computationally quite expensive and thus not applicable for this use case. Sun et al.^11^ introduced a fully convolutional neural network to predict the displacement between pairs of multi-modal image patches, without explicitly performing the optimizer and the similarity metric. They evaluated the method on US and CT liver slices (extracted from 3D volumes). The results were not satisfying for the real US and CT images.

In this work an example of such a navigation system for image-guided prostate biopsy is given: all transforms to fuse MR and TRUS image modalities were accomplished. In this work, the inter-modal registration MR/TRUS was replaced by an intra-modal registration of two additional 3D abdominal US (ab-3D-US) images. A quantitative system error analysis on a prostate phantom was evaluated and the target registration error (TRE) was estimated.

## Methods

### General workflow

**Figure 1:**
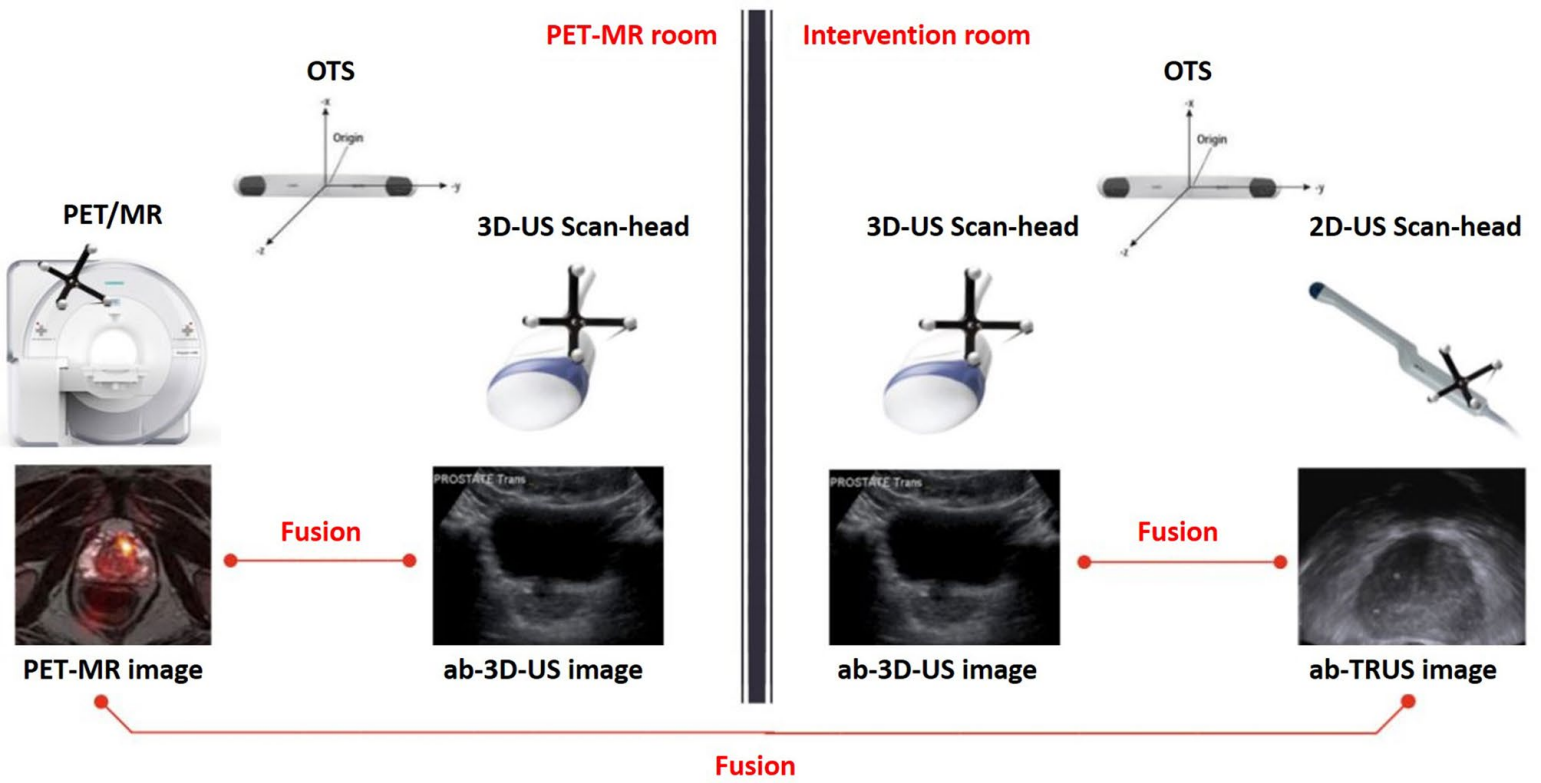
General workflow of the system: an ab-3D-US image is taken immediately after the PET/MR imaging. The transformation between the ab-3D-US and the PET/MR image modality can then be performed by simple calibrations using the OTS. The 3D-ab-US/3D-ab-US registration is replaced by a direct intra-modal ab-3D-US/3D-TRUS registration, to fuse the 3D free-hand TRUS with the pre-procedure PET/MR image.

The complete workflow (see Fig. 1) can be split into two parts: during the technical workflow, the pre-procedure image modality (i.e. MR, PET, SPECT, CT) is merged with an ab-3D-US volume. For this purpose, a number of calibrations must be carried out. These calibrations can be performed without any patient data and result in a transformation matrix which allows to merge the pre-procedure image modality with the optically tracked ab-3D-US. Furthermore, the intra-procedure (real-time) image modality has to be calibrated with respect to the OTS.

The patient specific workflow is decoupled from the calibration steps and includes pre-procedure patient imaging (i.e. MR, PET, SPECT, CT) and subsequent ab-3D-US scanning in a pre-procedure step. During this pre-procedure step, the additional US scan is performed within the regular pre-procedural imaging workflow. With the patient ab-3D-US taken, the images from the pre-procedure modality and the ab-3D-US can be fused avoiding any inter-modal registration. This fused image data set is then available for the intervention. Figure 1 shows an illustration of the procedures that result in an overlay of PET/MR and ab-3D-US. The intra-procedure step starts with another patient ab-3D-US from the same region of interest (ROI) corresponding to the pre-procedure ab-3D-US. Then, an ab-3D-US/ab-3D-US registration allows to link the pre-procedure PET/MR images with the intra-procedure ab-3D-US (and therefore also with the OTS in the intervention room). Consequentially, the pre-procedure and the (optically calibrated and tracked) intra-procedure images can be transformed into the same (OTS) coordinate system and be displayed by any kind of image overlay.

###  Feasibility study design

To prove the feasibility of such a system a prototype was built for prostate biopsy by fusing pre-procedure MR images with real-time 3D free-hand TRUS. This approach simplifies the general workflow by replacing the second ab-3D-US with the TRUS, i.e. the ab-3D-US/ab-3D-US registration is replaced by a direct ab-3D-US/3D-TRUS registration. The ab-3D-US scan would require an additional MR with the table coil for navigation/registration purposes. The usage of the table coil instead of the body coil might be preferable to avoid deformation artefacts. Therefore, the pre-procedure ab-3D-US can directly be registered to the TRUS which has been calibrated with respect to the OTS before. For intervention, the TRUS transducer has to be tracked and the MR image can be fused with the TRUS in real time.

The use of US close to a strong magnetic field was assessed by Hummel et al.^12^. To avoid damages of the MR gantry, the US device was kept outside the 200*mT* line where the force which acts on our US device (GE Voluson 6) was measured to be smaller than 20*N*. The 200*mT* line is distinctly marked on the floor around of the PET/MR gantry.

### Specifications

A GE Voluson E6 (General Electric Company, US^13^) ultrasound system with an RAB6-D convex transducer was used for the ab-3D-US imaging. The transducer, which works within a bandwidth of 2–8 MHz, consists of 192 piezo-electric elements. According to the manufacturer the US transducer had a resolution of axial 0.5 mm and lateral 2 mm.

The UroNav-MR/Ultrasound biopsy system (Invivo/Philips^14^) was used to obtain the 3D-TRUS images of the phantom. The system has the prostate triplane E14C4t transducer^15^, which works within a bandwidth of 4–14 MHz.

A fully-integrated PET/MRI system (Biograph mMR, Siemens Healthineers, 132 Germany) was used to scan the phantom. The sequence was a T1-MPRAGE-SAG-ISO, slice thickness 0.69 mm, echo-time 2.2 ms. The MR image has 1.06x1.06x1 mm^3^ spacing and 138x192x128 voxel dimension.

The tissue-equivalent prostate phantom with lesions (Model: 053-L, CIRS, Norfolk, VA^16^) was imaged with both the US and the MR system. The lesions had a radius of 5 mm as reported as smallest clinically significant tumor size in Gillies et al.^17^. The prostate (dimensions: 4x4.5x4 cm^3^) is made of Zerdine (low scatter); the background gel is similar to water with very little backscatter or attenuation.

The OTS is a Polaris Spectra (NDI, Waterloo, Ca^18^), which is assumed to provide a static accuracy of 0.25 to 0.35 mm within a volume of 1312 x 1566 x 1450 $mm^3$. It enables real-time 3D position and orientation tracking of tools which are composed of passive marker spheres.

### Calibration procedures

#### 3D US and TRUS calibrations

**Figure 2:**
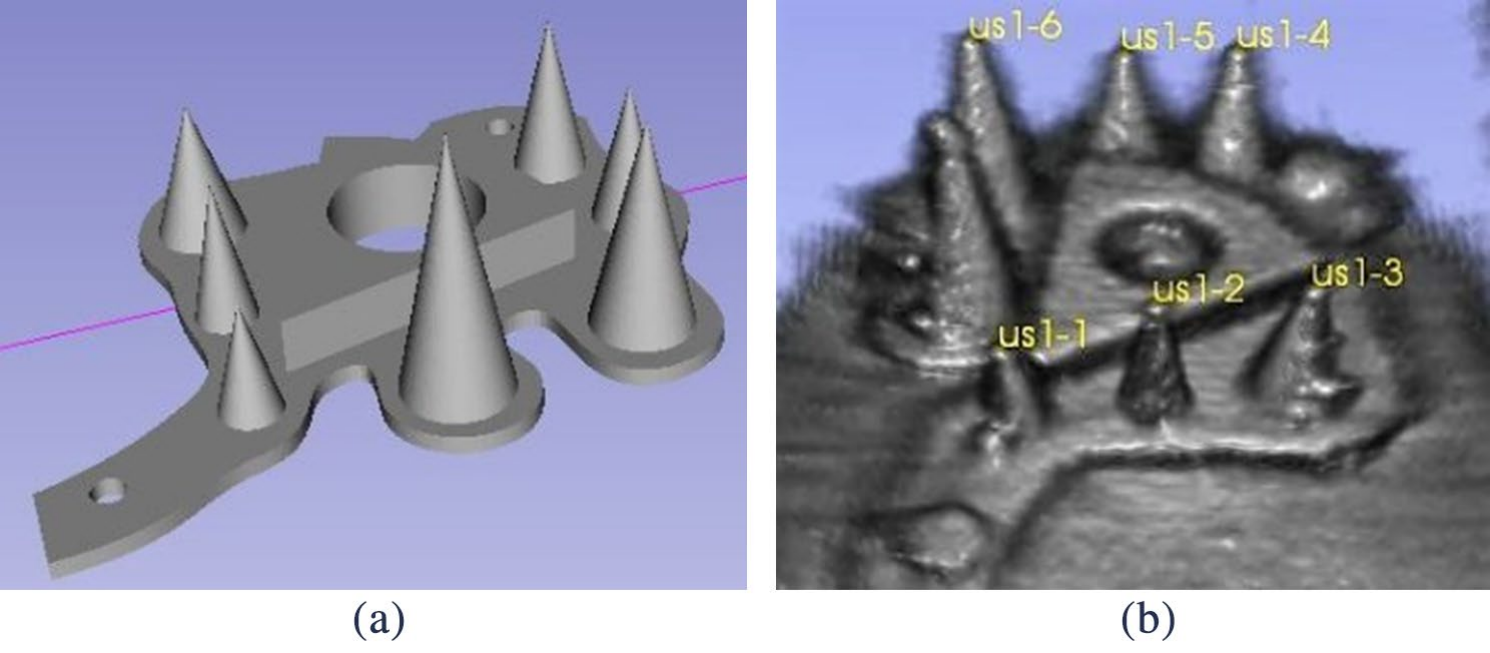
CAD model of the phantom for the 3D-US calibration (**a**) and its 3D US image on Slicer (**b**) with fiducials placed on the tips.

For the ab-3D-US calibration and evaluation, the same procedure as in Iommi et al.^19^ was performed. A positional sensor (i.e. an optical tracking tool) was mounted on the US transducer and the transformation ($T_{\small US}^{\small trans}$) from the image coordinate system (3D US image) to the coordinate system of the tracking tool (*trans*) was computed (spatial calibration). This was done by applying a point-to-point registration using fiducial markers (points) on a polylactide (PLA) multi-cone shape phantom. This phantom consisted of a 5 mm thick base plate with cones placed on top of the plate where the tips of the cones served as fiducials (Fig 2a).

The entire frame was placed in a tank made of perspex filled with water and fixed with screws. A tracking tool was rigidly attached on the tank representing the tank reference system. The calibration procedure was based on multiple images of the frame while changing position and orientation of the transducer. The coordinates of the seven cone tips in the US image coordinate system were determined by placing a cursor on each tip in the US image (Fig. 2b) while the coordinates of the tips with respect to the tank reference system were determined using a calibrated stylus. In order to minimize the jitter-error from the stylus measurements, each of the seven cone tips were measured 10 times adjusting the relative camera position 10 times. Seventy points, acquired on a second multi-cone phantom, from independent ten images taken from different camera positions were used to compute the TRE.

To increase accuracy we introduced an additional phantom calibration^19^. The coordinates of the cone tips were gathered in the coordinate system of the tank reference system as well as in the coordinate system of the CAD model of the phantom . This resulted in a transformation which was used to re-calculate the coordinates of the fiducials in the reference system from the more precisely measurable points in the CAD volume.

For the TRUS calibration a point-to-point registration was applied as described in the 3D Slicer tutorial^20^. The TRUS transducer was mounted in a fixed position upon an empty water tank and a calibrated pointer was swept within the US image field of view (FOV). Tracker data were recorded automatically and the corresponding image coordinates were determined by marking at the maximum intensity profile of the pointer tip on the image for certain time points. The list of fiducials (15 fiducials on the 2D-TRUS image) given in the two coordinate systems (i.e. the image and the OTS coordinate system) were then used for the point-to-point registration.

#### MR calibration

**Figure 3:**
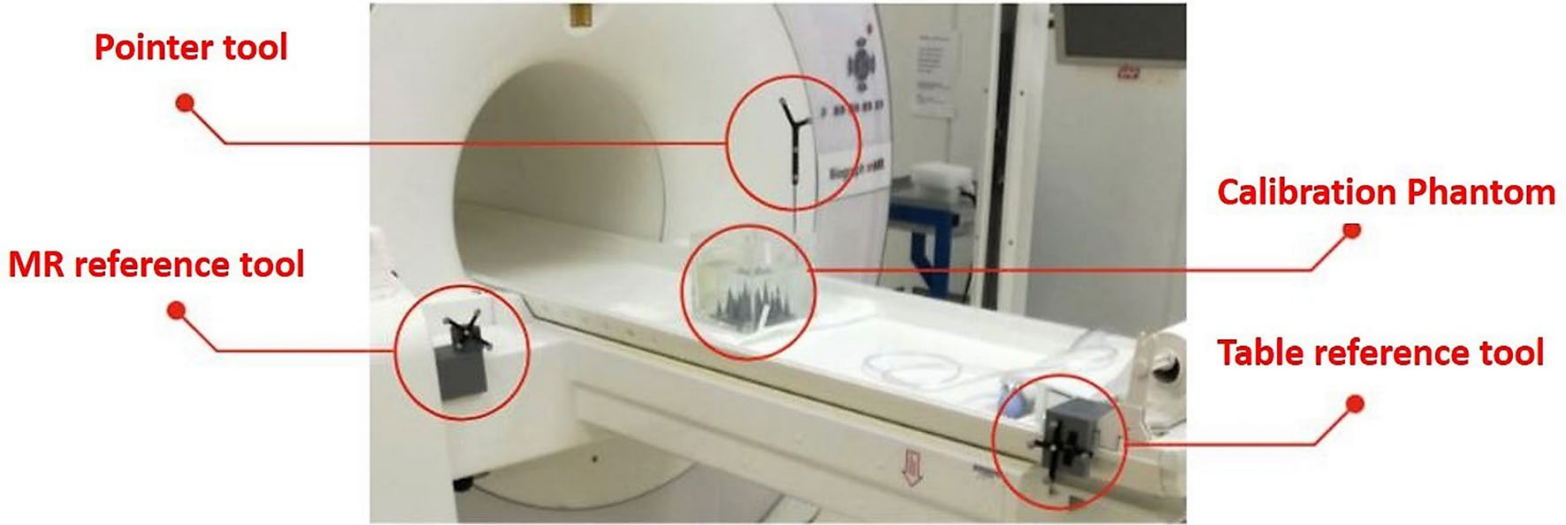
Set-up of the MR calibration. The coordinates of the calibraion phantom are measured respect to the MR reference tool with a calibrated pointer tool. This enables to determine the transformation $T_{\small MR}^{\small ORF_{MR}}$ from the MR-image coordinate system to the coordinate system of the optical tool mounted on the gantry of the MR.

The MR calibration was carried out by a point-to-point registration to get the transformation $T_{\small ORF_{MR}} ^{\small MR}$ between the OTS reference coordinate systems ($\small ORF_{MR}$) and the MR image (*MR*). To measure the coordinates of the phantom tips with respect to the OTS two optical tracking tools were mounted rigidly on the MR gantry and on the MR table (see Fig. 3, labeled as ’MR reference tool’ and ’Table reference tool’, respectively). As with the 3D-US calibration, the tips of a multi-cone phantom with 18 cones served as fiducials. Each tip-fiducial was collected five times, without moving the camera. As it is not possible to collect the fiducials with the stylus when the phantom is placed in the field of view of the MR, a table shift is required. The cone tips were first recorded with respect to the Table reference tool, and second, the OTS tracked the table shift inside the MR gantry (transformation between the $ORF_{TABLE}$ and the $ORF_{MR}$). The coordinates of the tips with respect to the MR coordinate system were determined from the MR image data set.

### Registration procedures and image fusion

#### MR/ab-3D-US registration

**Figure 4:**
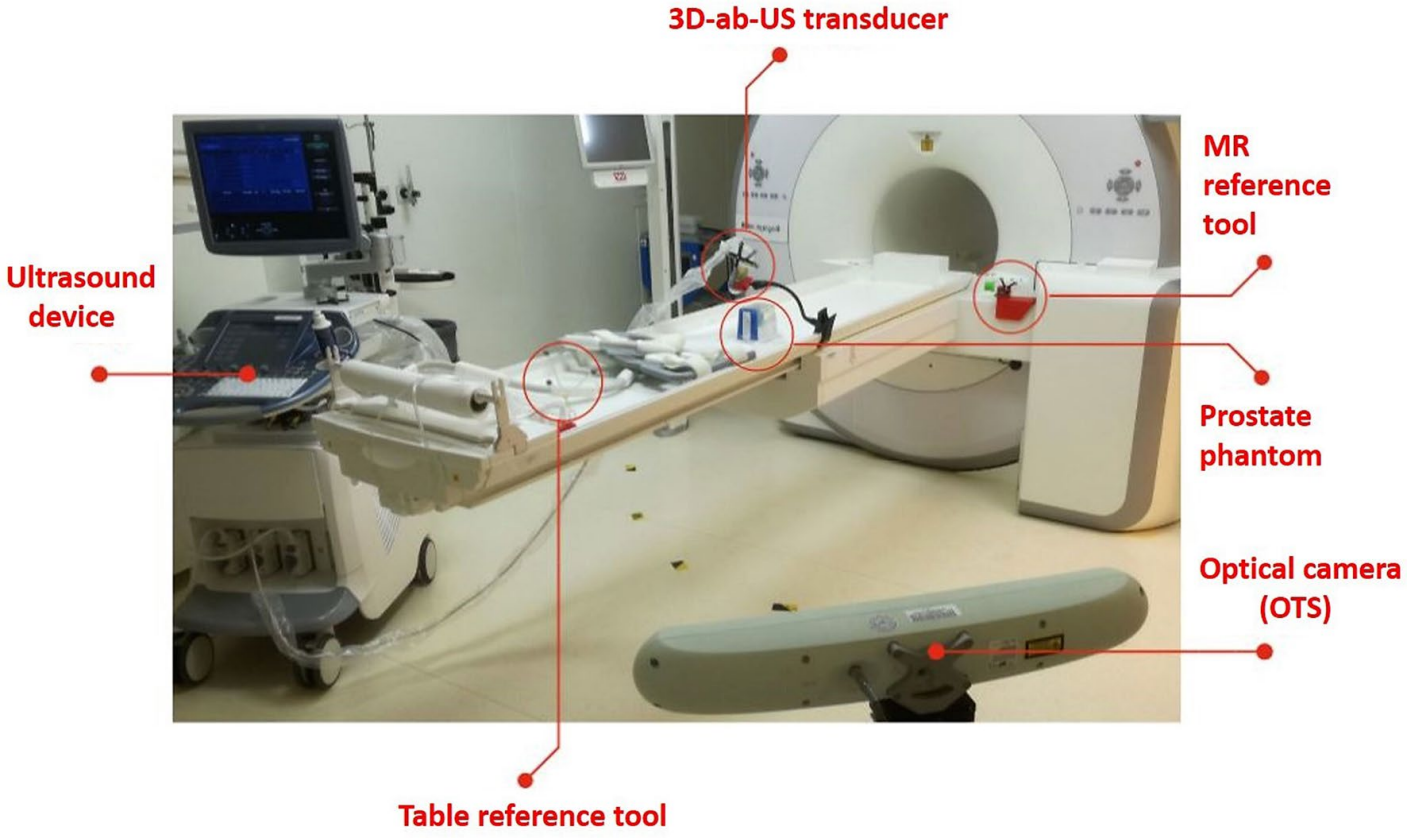
The principle set-up of the system. The phantom (the patient, respectively) is imaged with the ab-3D-US and then with the MR. US images can be linked with the MR using tracker data and US and MR calibrations. During intervention the 3D TRUS image of the phantom will be taken and a 3D-US/3D-TRUS registration augments the image with the MR volume.

The general system set-up is showed in Fig. 4. The OTS was positioned in the MR room and defined the global coordinate system. An ab-3D-US image was taken immediately after the MR scan. Since the 3D US transducer and MR were tracked and calibrated, the transformation from the MR to the US coordinate system did not require an inter-model registration but uses tracker data and calibration transformations only. Therefore, the image fusion between MR and the US coordinates systems can be calculated as:

1 $$\begin{aligned} T_{\small US}^{\small MR}=T_{\small ORF_{MR}}^{\small MR}\cdot T_{\small trans}^{\small ORF_{MR}}\cdot T_{\small US}^{\small trans} \end{aligned}$$

where $T^{\small ORF_{MR}}_{\small trans}$is the transformation between the US transducer tracking tool and the MR reference tool, given by the OTS.

*Table shift registration*

As a table translation between the MR scan and the ab-3D US acquisition will commonly be necessary (i.e. it is often not possible to acquire the ab-3D-US in the same position as the PET/MR), an additional sensor of the OTS (see Fig. 3 and 4, Table reference tool) was mounted on the MR table. This enabled to track a translation from the patient position during the MR scan to an arbitrary table position for the following ab-3D US acquisition. To determine the transformation of such a translation, the transformations $T^{ORF_{MR}}_{ORF_{TABLE}}$ and $T^{ORF_{TABLE}}_{ORF_{moved}}$ where calculated from the sensor readings before and after the table movements, where $ORF_{moved}$ repesents the reading of the table references tool after the movement.

The final transformation from the MR coordinate system to the ab-3D US coordinate system $T_{\small US}^{\small MR}$ is then given by

2 $$\begin{aligned} T_{\small US}^{\small MR}=T_{\small ORF_{MR}}^{\small MR}\cdot T_{\small ORF_{TABLE}}^{\small ORF_{MR}}\cdot T_{\small ORF_{moved}}^{\small ORF_{TABLE}}\cdot T_{\small ORF_{trans}}^{\small moved} \cdot T_{\small US}^{\small trans} \end{aligned}$$

Alternatively, the MR-calibration (see subsection 2.4) can be carried out by using $ORF_{TABLE}$ instead of $ORF_{MR}$ for the point-to-point registration. Consequently, formula 2 is then reduced to

3 $$\begin{aligned} T_{\small US}^{\small MR}=T_{\small ORF_{Table}}^{\small MR}\cdot T_{\small ORF_{moved}}^{\small ORF_{TABLE}}\cdot T_{\small trans}^{\small ORF_{moved}} \cdot T_{\small US}^{\small trans} \end{aligned}$$

where $T_{\small MR}^{\small ORF_{TABLE}}$ results from the MR calibration.

**Figure 5:**
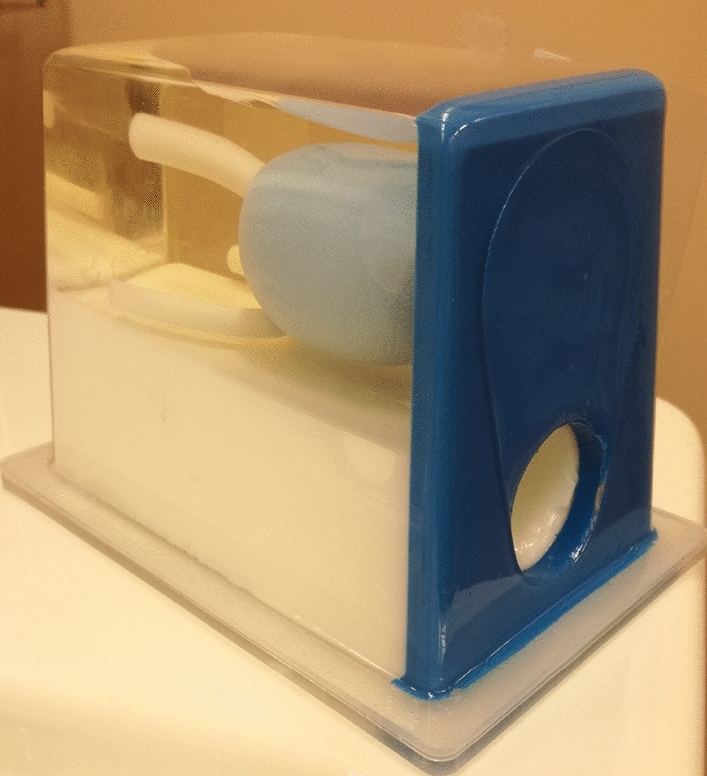
Tissue equivalent US prostate phantom with lesions.

The MR/ab-3D-US image fusion was tested using the multi-cone phantom and a tissue equivalent US prostate phantom with lesions^16^ (see Fig. 5). The procedure was performed two times in two independent tests, in which a total of five different 3D-US images were registered with the MR image by means of the transformations chain from equation 1 . The coordinates of the cones tips were determined in the MR image and in the ab-3D-US image manually. Finally, the mean TRE was calculated. With respect to the prostate phantom, the ab-3D-US images were acquired and registered with one MR image. The lesions in the prostate phantom were segmented in the MR image and ab-3D-US images using 3D Slicer. The mean Dice score, the Hausdorff distance and the mean boundary distance between the segmented lesions from the two overlaid image modalities were computed.

#### ab-3D-US/3D-TRUS registration

The images of the prostate phantom taken with the 3D-US abdominal transducer were registered with five 3D free-hand TRUS images. The images were masked manually. These reduced volumes mainly contained the prostate region and were registered with the registration tool in 3D Slicer by applying a ab-3D-US/3D-TRUS affine registration with Mattes mutual information metric^21^. This resulted in the transformation matrix $T_{\small TRUS}^{\small US}.$ The mean TRE between the centroids of the three segmented lesions was computed. The lesions in the 3D freehand TRUS and the ab-3D-US images were manually segmented and the mean Dice score with the Hausdorff distance and the mean boundary distance were computed.

#### MR/3D-TRUS image fusion

The 3D-TRUS images were merged with the pre-procedure MR images by transforming the points $P_{TRUS}$ from the 3D-TRUS images space to the MR images space by

4 $$\begin{aligned} P_{MR}=T_{\small US}^{\small MR} \cdot T_{\small TRUS}^{\small US}\cdot P_{TRUS} \end{aligned}$$

using the transformations described above. $P_{MR}$ indicates the corresponding point in the pre-procedure MR image space. Again, the mean Dice score, the Hausdorff distance and the mean boundary distance were calculated to evaluate the complete transformation. Additionally, the mean TRE between the centroids of the segmented lesions was computed.

## Results

### **3D US and TRUS calibrations**

The FRE for the 3D US calibration resulted to 0.87*mm* while the TRE was $1.0 \pm 0.48$ mm. The FRE for the 3D TRUS calibration resulted to 1.18*mm*.

### **MRI calibration and MRI/ab-3D-US registration**

**Figure 6:**
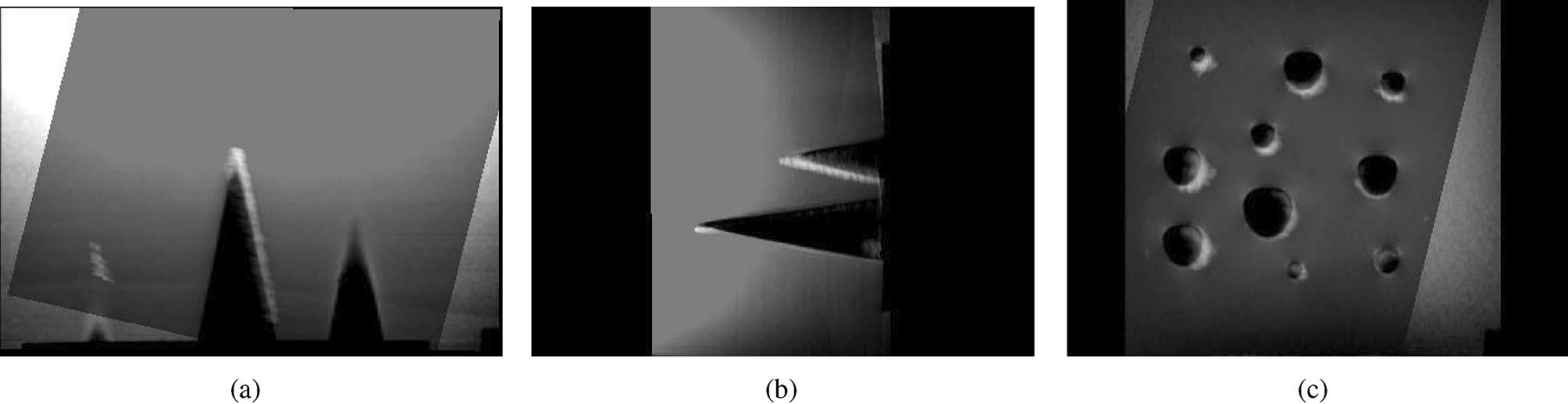
Overlaid images from the axial (**a**), sagittal (**b**) and coronal (**c**) planes with the registered MRI and ab-3D-US images of the multi-cone phantom. The cones are black in the MR image and the white in the US image. For this registration the TRE was calculated.

The FRE for the MRI calibration was 0.88*mm*. Figure 6 shows an example of the image fusion between the MRI and the ab-3D-US of the multi-cone phantom used for registration. The TRE of the MRI - 3D abdominal US registration for the cone shape phantom was found to be $1.81 \pm 0.31mm$. The mean Dice score between the segmented lesions resulted to 0.67. The mean of the Hausdorff distances between the segmented lesions was 3.19 mm. The average of the mean boundary distance resulted in 0.85 mm.

**Figure 7:**
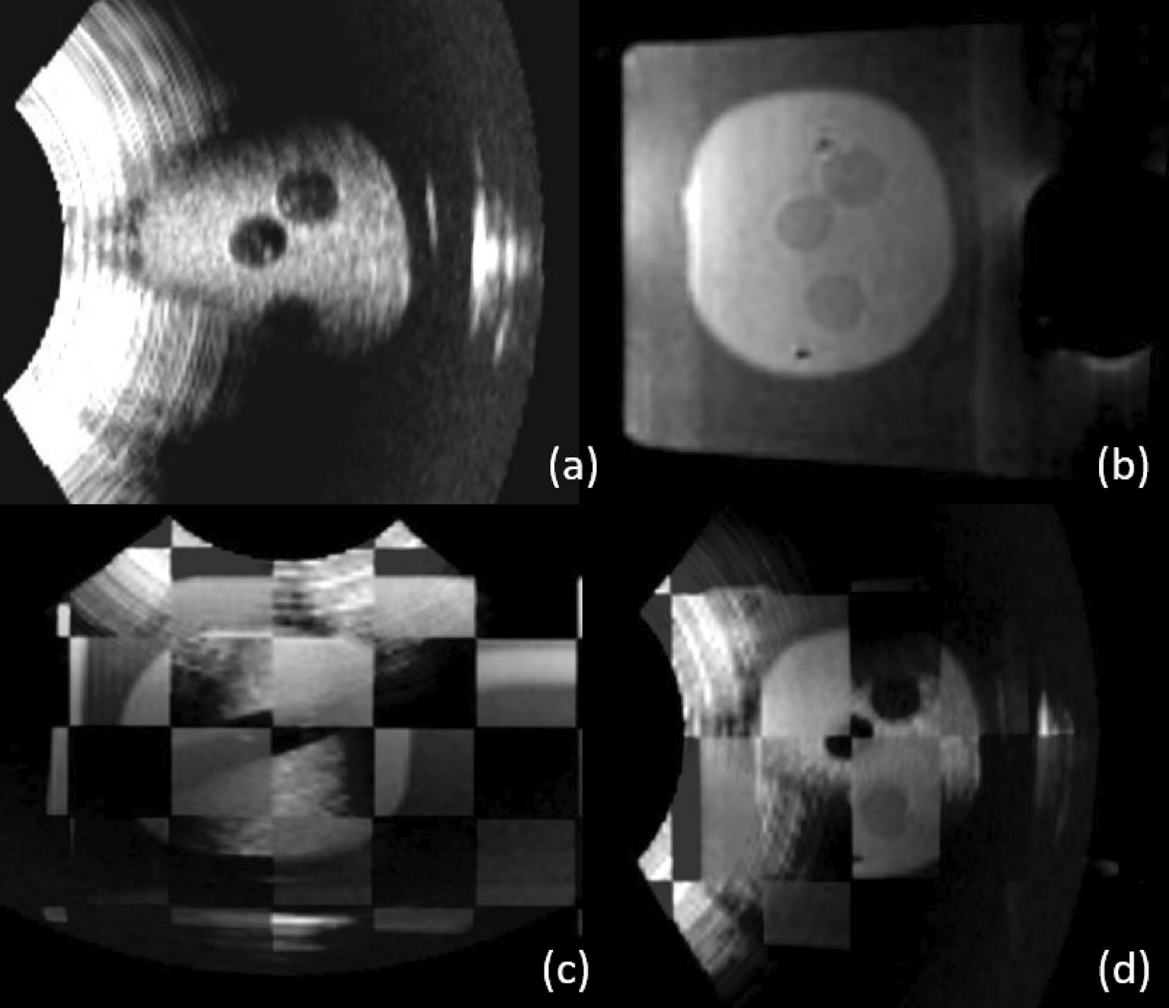
Sagittal views of the prostate phantom images from the ab-3D-US (**a**,**b**) and from the MR (**b**). Checkerboard images from from the coronal (**c**) and sagittal plane (**d**) from the registered MRI and ab-3D-US images of the prostate phantom. From this registration the Dice score was calculated from the segmented lesion.

Figure 7 shows an example of the overlaid registered MR and ab-3D-US images of the prostate phantom.

### **ab-3D-US/3D-TRUS registration**

The mean TRE between the 3D abdominal US and the TRUS images was $2.10\pm 0.2mm$ of five registrations after applying an affine registration. The mean Dice score between the segmented lesions from the ab-3D-US and the 3D-TRUS images was 0.73. The mean of the Hausdorff distances and average of the mean boundary distance between the segmented lesions were 2.29 mm and 0.74 mm, respectively..

### **MR/3D-TRUS registration**

**Figure 8:**
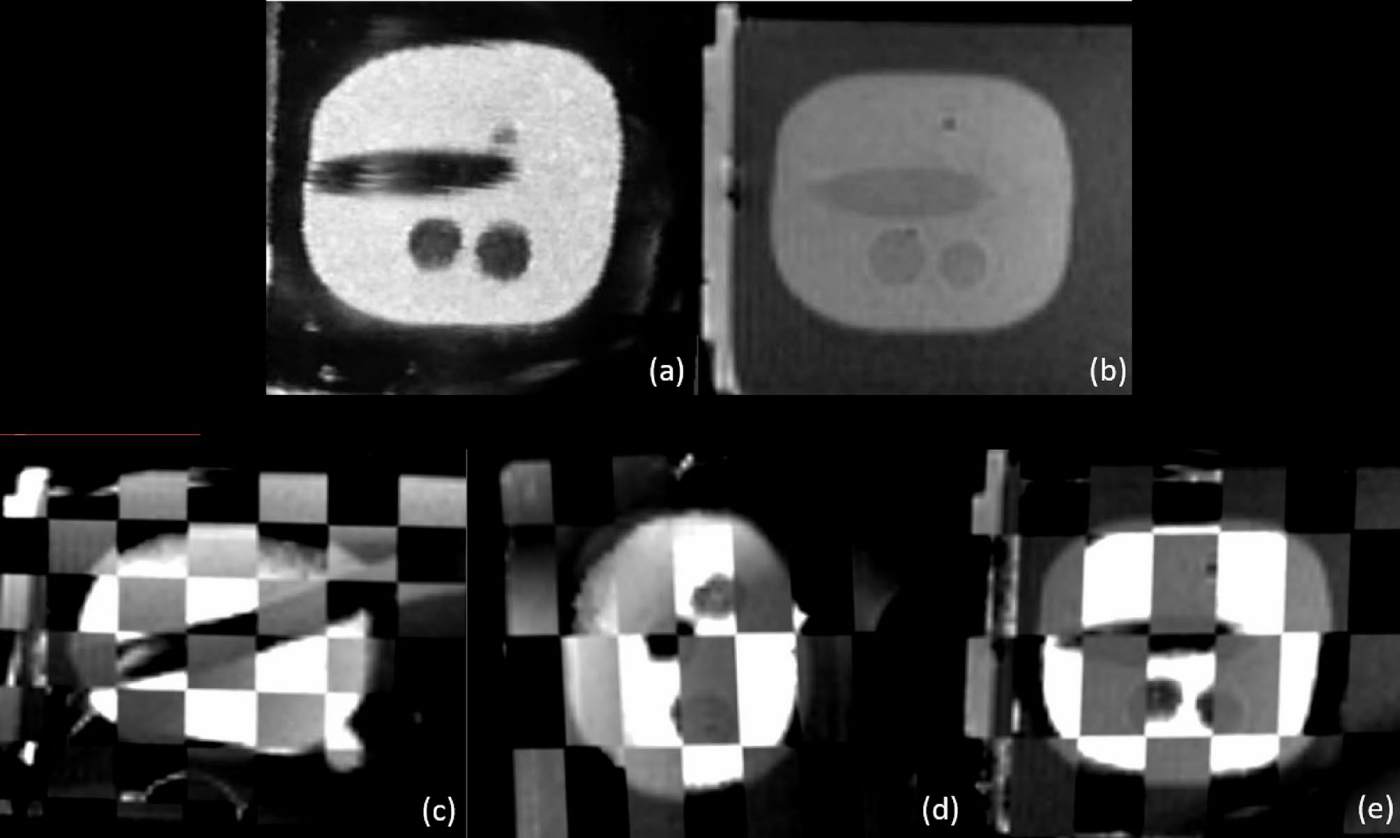
Coronal views of the prostate phantom images from the 3D-TRUS (**a**) and from the MR (**b**) and images from the axial (**a**), sagittal (**b**) and coronal (**c**) planes with the registered MRI and the 3D-TRUS images using the prostate phantom. From this registration the Dice score was calculated from the segmented lesion.

The mean Dice score between the segmented lesions from the MRI and the 3D-TRUS was 0.67 of five registrations. The mean TRE between the registered MR and 3D TRUS images was $2.52 \pm 0.65mm$. The mean of the Hausdorff distances between the segmented lesions amounted to 3.18 mm while the average of the mean boundary distance was 0.88 mm. Figure 8 shows an example of the image fusion between the MRI and the 3D-TRUS with the prostate phantom.

## Discussions

In this work a prototype of a navigation system which allows for image fusion of two arbitrary image modalities by replacing an inter-modal registration by an intra-modal 3D-US/3D-US registration and the use of an OTS is presented. Pre-procedure MR images were fused with 3D free-hand TRUS images for prostate biopsies on a phantom. A quantitative system error analysis was performed by assessing all calculated and measured transforms.

The error of the 3D US calibration was in the same range as in Iommi et al.^19^ where a TRE of lower than 1 mm was suggested. The error found for the complete transformation was comparable to the errors published by Zettinig et al.^4^. The evaluation of the Dice scores between the segmented phantom lesions (5 mm radius, clinically significant tumor size^17^) showed promising results and accuracy for the inter-modal registration between MR and TRUS images. In order to increase the registration accuracy, further studies could aim to minimize the jitter error from the OTS and the errors coming from the calibration steps.

In Hummel et al.^12^, to estimate the total error expected with patient data, a 3D-US/3D-US registration was evaluated considering the different patient positions during pre-procedure image acquisition (supine) and biopsy procedure (lateral). Three 3D US images of a volunteer were acquired at each of the two positions. The images were masked manually so that the remaining volumes mainly contained the prostate. The masked regions were then registered to each other applying a 3D-US/3D-US registration. In this experiment, a registration error of $3.7 mm \pm 1.1 mm$ was found compared to an error of $2.1 mm \pm 0.65 mm$ from our phantom experiments. Assuming a simple error propagation for linear transformations^22^, the total error from the MR to the TRUS should be around 3.9*mm*, which is acceptable considering clinically significant lesions with a diameter of 10*mm*^17,23^.

The presented system introduces an additional US device and might be bulky in a congested diagnostic room. Workflows, which include the acquisition of an additional prostate US scan in the pre-procedure room, have been already proposed by other groups^17,24^ to explore the advantages of intra-modal registration. In Cool et al.^24^, a pre-procedure 3D-TRUS volume was acquired at the time of the MR scan and registered with the MR image using manually identified anatomical landmarks. Nonetheless, the proposed navigation system implies the possibility to develop in the future a new prototype for trackerless biopsy. During the intervention, the TRUS could be repeatedly registered to the 3D abdominal US and the PET/MR images could be fused with the TRUS. For this repeated registration task, deformable registration algorithms based on mutual information and intesity based methods^25^ can be applied for the intra-modal US-US registration. Existing works showed the possibility to register the pre-procedure 3D-US volume with the intra-procedure 2D-TRUS image, by applying additionally motion compensation of the prostate in procedure room^17^. To speed up this registration step, recent artificial intelligence methods, based on convolutional neural networks (CNN), seems to be preferable. In fact, unsupervised methods are available for efficient and accurate intra-modality registration of 3D medical volumes which determine the linear and deformable parts in a single forward pass. Given a pair of scans, the registration field is computed by directly evaluating the function using the CNN model learned parameters. Recently, Balakrishnan et al.^26^ presented a fast learning-based algorithm for deformable, pairwise 3D medical image registration. Their CNN used a spatial transform layer to reconstruct one image from another while imposing smoothness constraints on the registration field. The proposed method did not require supervised information such as ground truth registration fields or anatomical landmarks. De Vos et al.^27^ developed a similar CNN framework to perform affine image registration and for deformable image registration, based on unsupervised training of the network. Li et al.^28^ proposed an adversarial learning framework for deep-learning-based deformable image registration with US images. The method showed promising results to register 3D US liver images for radio-frequency ablation for liver cancer.

Another potentiality of the proposed method is the development of a technology that enables ultrasound (US) guidance combined with fully automatic image fusion between high-resolution (CT, MR) and functional (SPECT, PET) images with common 2D-US imaging devices and off-the-shelf video cameras. The core technology of the proposed image guidance system is represented by the intra-modal 3D-US/3D-US registration procedure that allows for pre-operative image fusion (i.e. SPECT-CT, or PET-CT) as well as linking these pre-operative images with real-time image modalities (US). The 3D-US images could be reconstructed from 2D-US image sequences (know as freehand 3D US)^19^ where the 2D-US scan head is tracked with a video camera tracking system. Generally, such a navigation system can be designed to be used in wide range of clinical applications. In principle, such a free-hand 3D US could also be used instead of a 3D probe but experiments in^19^ have proved slightly better results with the 3D probe than with the freehand 3D US method.

Although the whole navigation system would work with an OTS alone, an electromagnetic system (EMTS) could be used in the intervention room to overcome the restriction of a free line of sight between camera and sensor. In this case, an electromagnetic sensor (such as Aurora, NDI^29^) will be mounted on the ab-3D scan head as well as on the TRUS scan head for tracking. This avoids limitations to the interventionists as they do not have to keep attention on the free line of sight needed for an OTS. A drawback of this alternative lies in the fact that the OTS tracking error is a magnitude lower than the error of the EMTS and the EMTS tracker measurements could also be distorted by the presence of metallic objects (Hummel et al.^30^). Nevertheless, the contribution of the tracker error to the overall error is small compared to other error sources and and An ETMS, used in a proper way^31^, would not affect the overall accuracy too much.

To enhance registration accuracy and to compensate for movements of the prostate between the pre-procedure and intra-procedure US scans, image masking is essential. In contrast to segmentation it is not necessary to determine the exact contour of the gland surface but to define a region of interest (ROI) where the organ can be found. After that, an automatic image registration based on CNN can be run to fuse permanently the 3D abdominal US images with the TRUS.

A fundamental challenge for navigation systems operating with preoperative images is deformation and organ movement. One can distinguish between deformations influencing the alignment of the MRI and the ab-3D-US which could affect the overall registration, and on the other hand deformations which occur in similar way in both ab-3D-US scans.

Deformations of the first kind caused by the MR body coil should be avoided if possible. One source of deformation might be the tightening/untightening of the MR body coil as used for our phantom study. If such problems with the body coil occur during patient studies, the built-in coil inside the patient table can be used alternatively instead of the external body coil. To quantify a possible reduction in image quality, the peak signal-to-noise ratio (PSNR) was calculated between MR phantom images using the abdominal and external body coil. The resulting PSNR was 25 dB.

The additional ab-3D-US will change the diagnostic workflow, as there will be a repeated MR with the ab-3D-US performed at that procedure. This additional MR will be acquired with the table coil to avoid possible deformation artefacts that might be caused by the body coil.

Organ motion might cause misregistrations if the motion is massively different in the two imaging procedures. An equivalent motion, e.g. caused by respiration or the heart beat should not cause considerable registration errors, given the images were recorded in the same phase of the motion which can be ensured by holding breath or by using an image/volume sequence and subsequent selection of the optimal image. Prostate motion induced by respiration is mainly found in situations with confined space as e.g. caused by immobilization shells^32^.

Another example of such an equivalent motion would be the application of the US transducer in a similar way in both the pre-procedure room and the procedure room, as discussed in^33^. As mentioned there these ab-3D-US images are only used to connect the OTS coordinate systems, wherefore an equivalent deformation would cancel out.

Nevertheless, the application of the transducer would likely cause only a moderate organ movement. In^34^ a median organ shift of less than 1 mm was found due to the application of the abdominal transducer. The deformation of the prostate itself was found to be negligible^33^. Nevertheless, such small motions and/or deformations could be compensated by applying motion compensation techniques as described e.g. by Marstal^25^, by Gendrin et al.^35^, or by Yang et al.^36^.

## Conclusions

The presented feasibility study has proven that our image fusion navigation system can be used to replace inter-modal registration with intra-modal registration by applying additional abdominal 3D US. The error from the full transformation chain from phantom experiments was in an acceptable range for the use in clinical applications such as prostate biopsy.
